# Technical Feasibility of a Multimodal Electro-Optical and Infrared Imaging Pipeline for Postoperative Wound Assessment After Total Knee Arthroplasty: Formative Evaluation Study

**DOI:** 10.2196/99244

**Published:** 2026-10-08

**Authors:** Tej S Ranade, Emily Hu, Kadambari Beelwar, Sandeep Boddeda, Praharsha Mulpur, Adarsh Annapareddy, A V Gurava Reddy

**Affiliations:** 1 Truss Health, Inc. Las Vegas, NV United States; 2 Sunshine Bone and Joint Institute Krishna Institute of Medical Sciences Begumpet, Telangana India

**Keywords:** AI, knee arthroplasty, periprosthetic joint infection, postoperative complications, infrared imaging, remote monitoring

## Abstract

**Background:**

Early detection and monitoring of wound-related complications after total knee arthroplasty (TKA) are critical to optimize outcomes and preserve implants. Conventional postoperative monitoring relies on in-person assessment and patient-reported symptoms, which may delay recognition. Noncontact imaging acquired in the clinic or at home offers a potential route to earlier and more consistent assessment.

**Objective:**

This study aimed to evaluate the technical feasibility and performance of a multimodal AI system integrating electro-optical (EO) and infrared imaging for postoperative wound assessment following TKA and to explore clinical factors associated with early complications.

**Methods:**

We conducted a single-center prospective cohort study of 749 patients undergoing primary TKA. Patients underwent standardized multimodal data collection at scheduled postoperative visits. EO-based models localized surgical landmarks and segmented complication-oriented tissue states; a fine-tuned vision-language model provided protocol verification and contextual visual reasoning. EO landmarks mapped to thermal space via deterministic alignment, followed by infrared region-of-interest segmentation and thermal classification. Each module was evaluated on a defined held-out set. The thermal classifier was assessed out-of-fold under 5-fold cross-validation with folds grouped by patient, in knees imaged on postoperative day 14 or later. Discrimination for clinician-adjudicated infection-related complications served as the primary outcome. Comorbidities and baseline laboratory values were explored for associations with complications.

**Results:**

Among 749 patients, 26 (3.5%) had a clinician-adjudicated infection-related complication. On held-out evaluation, the EO landmark detector reached a sensitivity of 0.967 (95% CI 0.954-0.979) and positive predictive value of 0.955 (95% CI 0.940-0.970), and the wound-tissue segmentation model reached a sensitivity of 0.845 (95% CI 0.833-0.857) and positive predictive value of 0.759 (95% CI 0.744-0.772). Infrared region-of-interest extraction isolated the surgical field with a mean intersection-over-union of 0.985 (SD 0.008; 95% CI 0.984-0.986) across 178 held-out patients. The trained thermal classifier discriminated infected scans with an area under the receiver operating characteristic curve (AUROC) of 0.978 (95% CI 0.959-0.992) in out-of-fold, patient-grouped cross-validation. At a representative operating point, sensitivity was 0.808 (95% CI 0.65-0.92), specificity 0.974 (95% CI 0.96-0.99), positive predictive value 0.618 (95% CI 0.49-0.78), and negative predictive value 0.990 (95% CI 0.98-1.00). Exploratory analyses showed nominal associations of chronic kidney disease and diabetes mellitus with postoperative complications that did not remain significant after multiplicity correction.

**Conclusions:**

In this formative study, a multimodal EO and infrared imaging pipeline demonstrated technical feasibility for postoperative wound assessment following TKA, providing noncontact discrimination of infection-related complications that compared favorably with established adjuncts. This work provides proof-of-concept for multimodal imaging-based assessment and defines the technical requirements for the prospective, multicenter external validation needed to establish clinical utility.

## Introduction

Total knee arthroplasty (TKA) is among the most common orthopedic procedures worldwide, with procedure volumes in the United States projected to reach 1.26 million annually by 2030 [1]. Despite high success rates, postoperative complications, particularly periprosthetic joint infection (PJI), remain significant drivers of morbidity, readmission, and cost [2-4]. PJI accounts for approximately 24%-36% of revision procedures in high-volume centers [2-4] and is associated with substantial morbidity and costs [5]. We combined recent point estimates for nonoverlapping end points, including PJI by 90 days (0.80%), symptomatic venous thromboembolism by 30 days (1.19%), 30-day major or postdischarge bleeding (3.43%), and early hematoma (0.24%-0.50%) and computed the union of events probability under independence, yielding an estimated union probability of 5.57%-5.82% [2,5-12].

Early recognition of postoperative complications after TKA remains challenging. Current surveillance relies on in-person assessment, wound inspection, serologic markers, and patient-reported symptoms [6,7]. These methods detect complications only after clinical manifestation, at which point infection burden and local tissue damage may already be substantial [8,9]. Evidence suggests that earlier detection improves outcomes [13,14], highlighting the need for scalable monitoring strategies that can identify postoperative complications at an earlier stage, including in outpatient and remote settings. Postoperative complication rates following TKA vary across sociodemographic groups, including differences according to sex, socioeconomic status, and comorbidity burden [15,16]; whether AI-based surveillance systems perform equitably across these groups remains to be established.

Advances in computer vision have enabled automated wound assessment using electro-optical (EO) imaging, with recent studies demonstrating its ability to localize surgical incisions and classify infection-related surgical-site abnormalities [11,12,17]. Infrared thermography has also emerged as a noninvasive technique for monitoring surgical-site healing following TKA [18-22]. Normal knees show a transient postoperative temperature elevation that resolves by 3 months, whereas chronic PJI is associated with persistent thermal asymmetry [23-25]. Prior thermographic studies after TKA have, however, been limited in scale, with a recent meta-analysis that pooled 318 patients across 10 studies [26]; larger investigations of postoperative skin temperature have relied on single-point infrared thermometry, which provides only tens of measurements per patient and cannot support spatial analysis [27]. Incorporating infrared radiometry alongside EO imaging may therefore enhance early detection by combining structural and physiological features.

The primary aim of this study was to develop and evaluate the technical feasibility of a multimodal AI system integrating high-fidelity EO and infrared imaging for postoperative wound assessment after TKA. As a secondary exploratory objective, we examined baseline clinical and laboratory factors associated with early infection-related complications. We hypothesized that multimodal integration would provide complementary information that could improve the characterization of post-TKA wounds compared with single-modality approaches.

## Methods

### Study Design

This was a prospective, single-center cohort study of adults aged ≥18 years undergoing TKA by fellowship-trained arthroplasty surgeons at a high-volume orthopedic hospital in India between January 2025 and May 2025. Baseline demographic, comorbidity, and laboratory data were collected at the time of enrollment. Demographic variables were age and sex; procedural variables were surgical approach (conventional or robotic-assisted TKA) and operative laterality (right, left, or bilateral). Baseline laboratory values were hemoglobin, serum creatinine, sodium, and potassium. Documented comorbidities included hypertension, diabetes mellitus, thyroid disease, coronary artery disease, and chronic kidney disease, among others. These baseline characteristics are summarized in Multimedia Appendix 1. Predefined exclusion criteria were bleeding disorders (eg, hemophilia), rare skin-related genetic disorders, prior revision knee arthroplasty, prior hip arthroplasty, and withdrawal of consent.

### Recruitment

Participants were recruited from patients scheduled to undergo TKA or who presented for TKA at the study site during the predefined enrollment period. Potential participants were assessed for eligibility according to the study inclusion and exclusion criteria. All eligible patients who expressed interest in participation were screened by the study staff, and participants were sequentially enrolled as they became available throughout the enrollment period. Sample size was determined by enrollment capacity during the defined study period rather than prospective power analysis.

### Ethical Considerations

The study was approved by the Institutional Ethics Committee (KIMS/IEC-RHR/2025/06/05) and conducted in accordance with the Transparent Reporting of a multivariable prediction model for Individual Prognosis Or Diagnosis (TRIPOD) guidelines for multivariable prediction models [28] and the Declaration of Helsinki. All procedures adhered to World Health Organization (WHO) Global Guidelines for the Prevention of Surgical Site Infection and National Institute for Health and Care Excellence (NICE) guidelines NG125 recommendations for postdischarge wound care [29,30]. This study is also reported in accordance with guidelines for reporting of AI-based research in orthopedic surgery research, published by Wyles et al [31]. All participants provided written informed consent before enrollment. To protect privacy and confidentiality, all study data were fully anonymized before analysis, ensuring no personally identifiable information was included. No financial compensation or other incentives were provided for participation, as the study was conducted on a voluntary basis. Finally, no identifiable images or personal data from participants are included in the study dataset.

### Longitudinal Imaging Data Collection

Paired infrared and EO data were acquired at postoperative visits for postoperative days 1, 15, 24, and 30, corresponding to routine clinic and physiotherapy appointments. In practice, acquisition timing varied with patient attendance, and all analyses used each scan’s recorded postoperative day rather than the nominal visit day. Imaging used a custom handheld device integrating an Intel RealSense D405 EO camera (640×480-pixel color) and a Seek Thermal Mosaic Core long-wave infrared radiometric sensor (7.8-14 µm; 240×320 pixels). Standardized acquisition was performed both during scheduled clinic visits and at patients’ homes during physiotherapist visits, capturing performance across clinic and in-home settings.

Thermal acquisition followed medical thermography guidance: emissivity ε≈0.98 with ambient reflection compensation, orthogonal framing, and consistent standoff distance, in accordance with IEC 80601-2-59 and ASTM E1933/E2847 [32-34]. Imaging was performed no sooner than 6 hours after any local icing or external heat application to the operative knee. During physiotherapy visits, data were captured before the therapy. At each postoperative visit, the treating clinician determined whether a complication was present based on examination and the clinical record. Complications were categorized a priori into 3 types, including infection-related wound dehiscence (defined as wound opening attributable to underlying infection), hematologic events (defined as dehiscence with visible oozing blood and purulent discharge), and confirmed PJI without visible dehiscence. Complication status was adjudicated based on visible signs of infection at the surgical site, specifically purulent discharge and open wound.

### Dataset and Development Sets

Of the 1065 patients screened, 749 (70.3%) met the inclusion criteria; the remainder were excluded for incomplete medical records, data inconsistency or irregularities, inability to complete follow-up, or nonadherence to the study protocol. Thirty-four infection-positive patients were identified during follow-up, of whom 8 were excluded on predefined data-completeness grounds: one had a missing baseline serum potassium value, and 7 did not return for follow-up assessment, and confirmation of healing was not obtained through attendance or telecommunication attempts. This yielded 26 adjudicated infection-related complications in the analytic cohort (26/749, 3.5%). Of these, 25 out of 26 (96%) presented with wound dehiscence attributable to infection, of which 1 out of 26 (4%) additionally exhibited oozing blood with purulent discharge meeting the definition of a hematologic event. One patient (1/26, 4%) had confirmed PJI without visible dehiscence. The 749 patients contributed 2606 co-registered radiometric infrared and EO image pairs (84 complication and 2522 noncomplication pairs).

### Thermal classifier

#### Development Cohort and Cross-Validation

Development used radiometric data acquired at postoperative day 14 or later, comprising 409 patients (26 with infection-related complications and 383 controls) who contributed 1323 scans. This window was chosen because scan-level AUROC was 0.670 in the first postoperative week and exceeded 0.90 thereafter; within it, the number of scans available per knee was not itself predictive (area under the curve [AUC] 0.466), indicating that follow-up intensity did not act as a proxy for outcome. Separately, postoperative day was not used as a direct predictor, because complication-affected knees tended to be imaged later in the postoperative course, and day-of-scan would otherwise encode imaging schedule rather than pathology; thermal descriptors were instead expressed as deviations from expected uncomplicated healing at the same postoperative day, with the normative reference estimated from control scans in the training folds only. The classifier was trained and evaluated by 5-fold cross-validation with folds grouped by patient, so that both knees of a patient and all of that patient’s scans were assigned to a single fold. Every reported metric in this study was calculated out-of-fold; each patient was scored only by models fitted without that patient’s data, and results were averaged over 3 random seeds.

#### Task Framing

The classifier was framed as a combined detection and prediction task in which a knee was labeled positive if it developed a complication at any point during follow-up. Every scan of a complication-positive knee, including scans acquired before clinical onset, was assigned to the positive class, reflecting the intended clinical use in which any available scan should contribute to flagging an at-risk knee. Complementary learners were additionally trained under a stricter labeling scheme in which only scans acquired during a clinically present complication were positive; in this scheme, pre–onset scans were withheld from the training objective but were still scored at inference, so their observed values contributed to the patient-level aggregate and no score was imputed.

#### Inputs and Feature Representations

Each thermal scan was cropped to the region of interest (ROI) established by the EO pipeline, expanded by an 8% margin, and resized to 224×224 pixels so that imaging distance and framing did not influence the analysis. Each scan was encoded in 2 forms: as images, for convolutional analysis, and as a set of quantitative descriptors, for feature-based analysis. Both representations were derived from the radiometric thermal matrix within the ROI. Three complementary image encodings were used. The first was a 3-channel physics encoding comprising the thermal excess over ambient, the local SD over a 5×5 neighborhood, and the gradient magnitude. The second was a color map rendering of the thermal matrix, per-image minimum-maximum normalized. The third was a self-normalized encoding in which every channel was referenced to the knee’s own median and IQR, rendering it invariant to uniform shifts in scene temperature. The quantitative descriptors were physics-based thermal features organized into families: temperature and Stefan-Boltzmann radiant-exitance statistics, spatial gradient and local variability, the morphology and extent of localized hot regions, multiscale texture energy from a scattering transform across 4 spatial scales, band contrasts oriented along the incision axis, and each scan’s deviation from the patient’s own baseline, defined from the earliest complication-free scan and from expected uncomplicated healing at the same postoperative day.

#### Model and Training

Complementary learners were trained on these representations. Convolutional neural networks were trained on each of the 3 image encodings under each of the 2 labeling schemes; each used a classification head (a dropout layer followed by a linear layer) and was optimized with AdamW (learning rate 2×10^-4^ and a weight decay of 10^-4^) under a cosine schedule for 25 epochs at a batch size of 32, with class-balanced sampling to account for the low prevalence of complications and a cross-entropy objective. Augmentation was limited to horizontal reflection, translations of up to ±12 pixels, and scale changes between 0.92 and 1.08; vertical flips and rotations were not applied, as the images have a fixed anatomical orientation. Horizontal-flip test-time augmentation was used at inference. Two additional learners operated on the quantitative descriptors: a self-supervised image-embedding model reduced by principal component analysis and classified by logistic regression, and a logistic-regression model on the physics descriptors with postoperative-day normative scoring. The learners’ patient-level scores were combined by equal-weight rank averaging, a parameter-free step with no combination layer fitted to the labels, and each knee’s final score was calculated as the mean of its scores across scans.

#### Confound Handling

Ambient temperature differed between the complication and control groups (27.4 °C versus 30.0 °C); because this difference was already present in scans acquired before clinical onset, it reflected the acquisition setting rather than the outcome. It was controlled in 3 ways: a self-normalized, ambient-invariant encoding that references every value to the knee’s own distribution so that a uniform shift in scene temperature cancels by construction, which as a standalone model reached an AUC of 0.950; measurement of each learner’s dependence on ambient temperature, which was low for the image-based learners; and regression of ambient temperature out of the patient-level score using control patients only, which yielded an AUC of 0.963, a conservative estimate of performance attributable to thermal pathology rather than to acquisition conditions.

#### Thermal Region-of-Interest Segmentation

Development used 2606 radiometric scans, partitioned into 2203 training scans and 403 validation scans. Thermal scans were labeled to create the binary segmentation masks of the operated knee as visible in the radiometric heatmap. The masks were used as ground truth. Training and validation were separated at the patient level so that no patient contributed images to both partitions, and the decision threshold was fixed on the training set before evaluation.

#### EO Models

The anatomical landmark detector, the fine-tuned vision-language model (VLM), and the complication segmentation model were developed on cohort EO images expanded with data augmentation and integrated with publicly available datasets. Offline augmentation was performed using feature pasting, cross-feature mixing, background substitution and drift, and morphological operations was applied on the fly for training only and was excluded from all validation sets, which comprised unaltered images. Offline augmentation used no image generative AI (GenAI) tools and was done manually using Python (Python Software Foundation) and image-editing tools (Figure 1).

The landmark detector used 8635 training and 426 validation images, containing 914 annotated objects, at a fixed, untuned confidence threshold of 0.5. The dataset comprised 7 classes, including bandage, feet, hand, healed incision, incision, leg, and leg with bandage. A bounding box was created for each label as the ground truth. The validation set was held-out from training and screened against the full training set by perceptual hashing, with no validation image falling within a near-duplicate threshold of any training image. The VLM was fine-tuned on a multilabel corpus of EO wound images. Training annotation counts were 1379 infections, 1052 blood presence, 728 purulent discharge, 704 open wounds, 516 discharge or exudate, and 283 cellulitis instances, within a broader set of 17,712 images containing a surgical site. The held-out validation set contained 152 infection, 132 blood presence, 68 purulent discharge, 60 open wounds, 56 discharge or exudate, and 44 cellulitis-positive instances within 1956 surgical-site images. The validation set did not contain any augmented images. The complication segmentation model used 37,062 training and 2003 validation images, partitioned at the image level, and incorporated publicly available open-wound datasets. The model was trained on the segmentation of 4 classes, namely, open wound, granulation, infection-associated exudate (IAE), and necrosis. IAE is the superclass representing a single label for the segmentation of purulent discharge.

**Figure 1:**
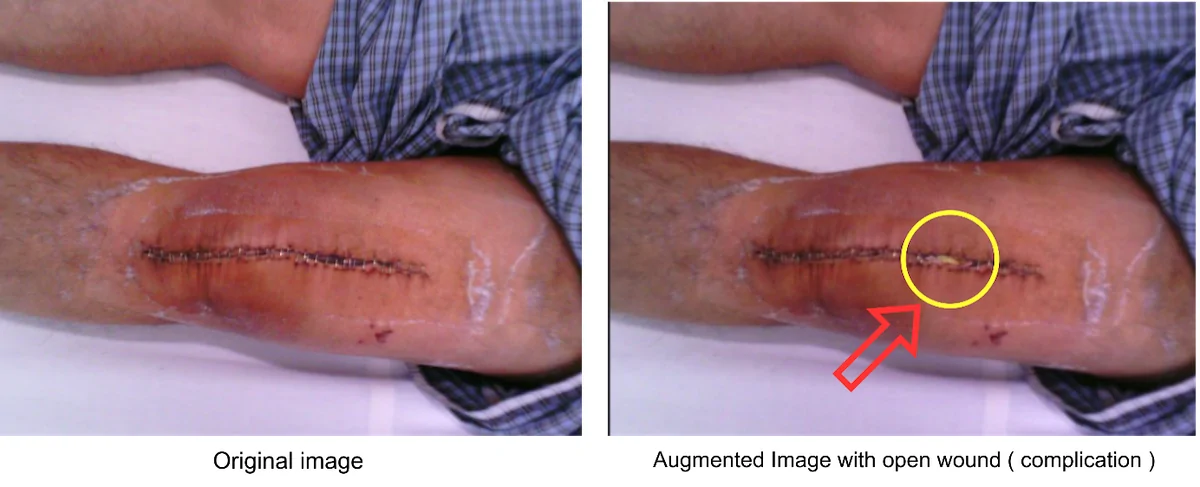
Illustrative offline data augmentation of an electro-optical wound image. Left, an original electro-optical frame from the study device showing an open postoperative wound; right, a representative augmented derivative produced by the manual compositing pipeline (feature pasting, cross-feature mixing, background substitution, and morphological operations).

### Image Annotation

Ground-truth image-level annotations were produced by 2 clinicians, a licensed physiotherapist and a fellowship-trained orthopedic surgeon, using a standardized label set comprising 6 categories, including infection, blood presence, purulent discharge, open wound, discharge or exudate, and cellulitis. Labels were applied at the surgical-site level to each EO image. To ensure diagnostic accuracy, all infection labels were cross-referenced against contemporaneous clinical records, including physical examination findings and serological markers of infection; where clinical record findings conflicted with the image-level annotation, the clinical record superseded the annotator label, and the annotation was updated accordingly. All annotations were reviewed for completeness and consistency by the principal orthopedic investigator prior to inclusion in the training dataset. Wound pixel-level segmentation and anatomical landmark labeling were performed separately by the research team under the supervision of the principal orthopedic investigator.

### Data Analysis

#### General Approach

All statistical tests were 2-tailed with α=.05. Continuous variables were assessed for normality with the Shapiro-Wilk test and summarized as mean (SD) and 95% CI or, when nonnormal, as median (IQR); proportions are reported with numerators, denominators, and Wilson 95% CIs. *P* values are reported to 2 or 3 decimal places without a leading zero, and as *P*<.001 below that threshold. Multiplicity was controlled with the Benjamini-Hochberg false discovery rate (reported as q) as the primary correction, with Holm-Bonferroni applied for strong family-wise error control.

#### Cohort Analyses

Two-group comparisons of continuous variables used the Mann-Whitney *U* test with the Cliff delta effect size, or the Student *t* test with the Hedges *g* effect size, when normality was satisfied; sensitivity analyses used the Welch *t* test and ordinary least-squares mean differences with HC3 robust standard errors. Multigroup comparisons used the Kruskal-Wallis test with epsilon-squared. Continuous-continuous associations used the Spearman rank correlation with 95% CIs. Categorical associations used the chi-square test with the Cramér V effect size or the Fisher exact test when any expected cell count was below 5. Binary exposure–outcome associations were expressed as odds ratios (ORs) with 95% CIs, with the Haldane-Anscombe correction applied only when a cell count was zero; continuous predictors of a binary outcome were analyzed using univariable logistic regression with prespecified scaling (per 10-year age, per 1-SD laboratory value). Test statistics, degrees of freedom where applicable, and effect sizes are reported with each result.

#### Model Evaluation

All performance metrics were computed on the held-out sets described under dataset and development sets. For the thermal classifier, discrimination was summarized by the area under the receiver operating characteristic curve (AUROC), and, given class imbalance, operating points were reported in clinical terms: sensitivity, specificity, and positive and negative predictive value, and the number needed to evaluate (defined as 1/positive predictive value). Single-figure operating points were obtained by nested threshold selection, with the decision threshold chosen on training patients and applied to held-out patients. CIs for classifier metrics were 95% percentile bootstrap intervals from patient-stratified bootstrap resampling (2000 resamples, preserving prevalence). Segmentation performance was summarized by the dice coefficient and intersection-over-union, reported per class and as macro- and micro-averages; detection performance by mean average precision under the Common Objects in Context (COCO) protocol; and region-of-interest segmentation by intersection over union. Segmentation and detection CIs were 95% percentile bootstrap intervals over images (1000 resamples). Machine learning–native measures are reported in full in Multimedia Appendix 2. Cross-validated results are reported as the mean across fixed random seeds.

#### Software

Statistical analyses were performed in Python 3.12 (*NumPy* 2.1.2, *pandas* 2.2.3, *SciPy* 1.14.1, and *statsmodels* 0.14.4). For data labeling, Roboflow (Roboflow, Inc) was used.

#### Proposed Multimodal EO and Infrared Imaging Architecture

We developed a sensor-agnostic pipeline that fuses EO imaging with infrared radiometry to convert postoperative data into structured, clinically interpretable indicators of wound status (Figure 2). The EO stream first establishes an invariant surgical coordinate frame. A transformer-based anatomical landmark model localizes the incision, operative limb, and exclusion zones so that all subsequent analysis is constrained to the correct field and remains comparable across visits. A semantic segmentation model then produces pixel-level masks for clinically actionable tissue states, including granulation, IAE, necrosis, and the aggregate wound field delineating infection-affected areas and open-wound conditions to support tracking and remote monitoring of the compromised site. A fine-tuned VLM provides contextual reasoning by verifying the anatomic plausibility of segmentations, identifying subtle patient-specific clinical signs, and generating protocol and quality control signals (standoff, orthogonality, and dressing presence) that safeguard the interpretation and detection of infection and blood presence as standalone adjunctive analysis.

Detections from the EO stream are transferred to thermal space by a deterministic EO-to-infrared projection that preserves geometry and restricts analysis to clinically relevant zones. Within these mapped regions, an infrared segmentation model isolates the radiometric ROI, suppressing environmental background while preserving landmark context. Finally, a radiometric classifier analyzes the thermal data and returns patient-level risk scores and spatial risk maps; its operating threshold can be selected out of sample to match clinical workload and risk tolerance, from a high-sensitivity rule-out setting to a high-specificity rule-in setting. EO models were developed on expert-annotated institutional images augmented with publicly available data and evaluated on held-out sets, and the thermal classifier was evaluated by patient-grouped cross-validation.

**Figure 2:**
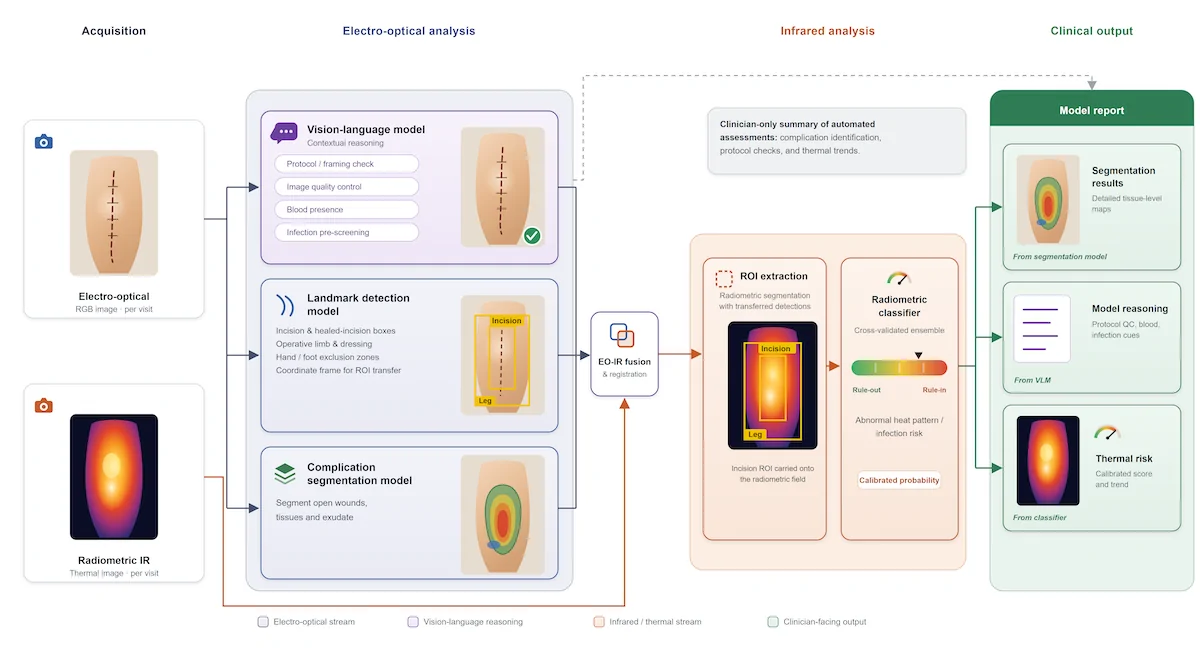
Architecture of the multimodal electro-optical (EO) and infrared analysis pipeline. EO: electro-optical; IR: infrared; RGB: red, green, and blue; ROI: region of interest.

## Results

### Patient Characteristics

The final cohort (Figure 3) had a mean age of 63.4 (SD 9.1) years (median 64, IQR 57-70), 70.1% (525/749) of patients were female, and 29.9% (224/749) were male. The surgical approach was robotic-assisted in 51.1% (383/749) of patients and conventional in 48.9% (366/749) of patients. Laterality was right knee in 327 (43.7%), left knee in 292 (39.0%), and bilateral in 130 (17.4%) of 749 patients. Mean baseline laboratory values were hemoglobin 12.3 (SD 1.5) g/dL (median 12.3 g/dL, IQR 11.2-13.3), creatinine mean 0.87 (SD 0.23) mg/dL (median 0.83 mg/dL, IQR 0.71-0.99), sodium mean 137.8 (SD 3.7) mmol/L (median 138 mmol/L, IQR 136-140), and potassium mean 4.4 (SD 0.6) mmol/L (median 4.3 mmol/L, IQR 4.1-4.6). Full baseline characteristics, including CIs and distributional statistics, are reported in Multimedia Appendix 1.

The most common comorbidities were hypertension in 521 patients (69.6%) and diabetes mellitus in 306 patients (40.9%). Additional comorbidities are detailed in Table 1.

**Figure 3:**
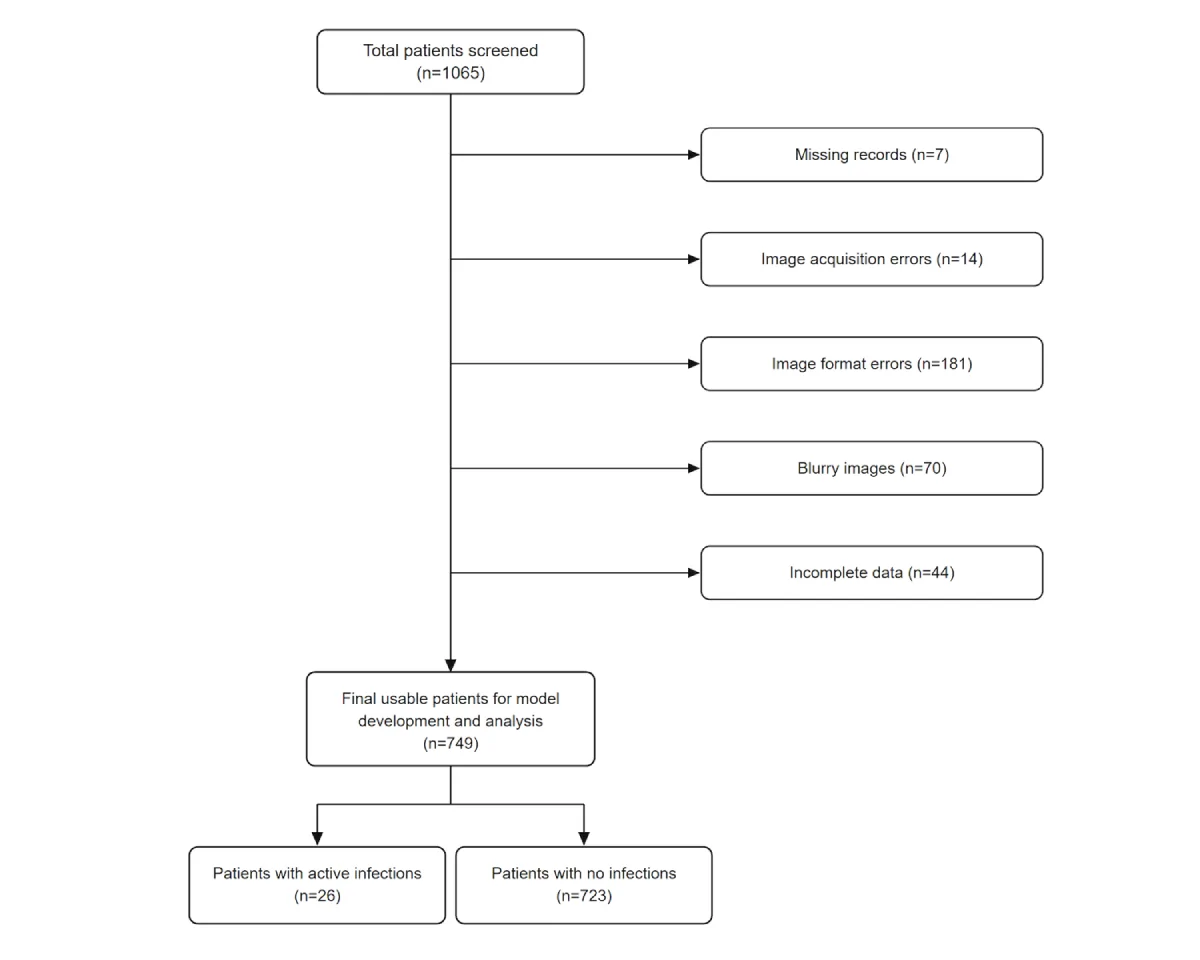
Cohort inclusion and exclusion.

**Table 1:** Comorbidity prevalence in the analytic cohort (N=749 patients). Values are n/N (%) with Wilson 95% CIs. Patients may have had multiple comorbidities; percentages therefore sum to more than 100%.

Comorbidity	n (%)	95% CI
Hypertension	521 (69.6)	66.2-72.7
Diabetes mellitus	306 (40.9)	37.4-44.4
Thyroid disease	168 (22.4)	19.6-25.6
Coronary artery disease	69 (9.2)	7.3-11.5
Asthma	39 (5.2)	3.8-7.0
Cerebrovascular accident	21 (2.8)	1.8-4.2
Chronic kidney disease	9 (1.2)	0.6-2.3
Epilepsy	7 (0.9)	0.5-1.9
Rheumatoid arthritis	7 (0.9)	0.5-1.9
Parkinson disease	4 (0.5)	0.2-1.4
Obstructive sleep apnea	2 (0.3)	0.1-1.0
Urinary tract infection	2 (0.3)	0.1-1.0
Bronchitis, coronary angiography, interstitial lung disease, and psoriasis	1 (0.1)	0.02-0.8

### Multimodal Model Performance

#### Landmark Detection Model

The transformer-based landmark detector was evaluated and reached a sensitivity of 0.967 (95% CI 0.954-0.979), a specificity of 0.986 (95% CI 0.981-0.990), a positive predictive value of 0.955 (95% CI 0.940-0.970), and a negative predictive value of 0.990 (95% CI 0.985-0.994). Mean average precision was 0.886 (95% CI 0.834-0.938) at an intersection-over-union threshold of 0.5 and 0.692 (95% CI 0.655-0.726) averaged across intersection-over-union thresholds from 0.50 to 0.95 (COCO protocol).

Detection of the surgical incision, the landmark to which all downstream analysis is anchored, was among the strongest, with an average precision of 0.940 at an intersection-over-union threshold of 0.5 and image-level sensitivity and positive predictive value of 0.958; localization of the operative leg and limb was similarly robust (Figure 4). Complete per-class detection metrics are provided in Multimedia Appendix 2.

**Figure 4:**
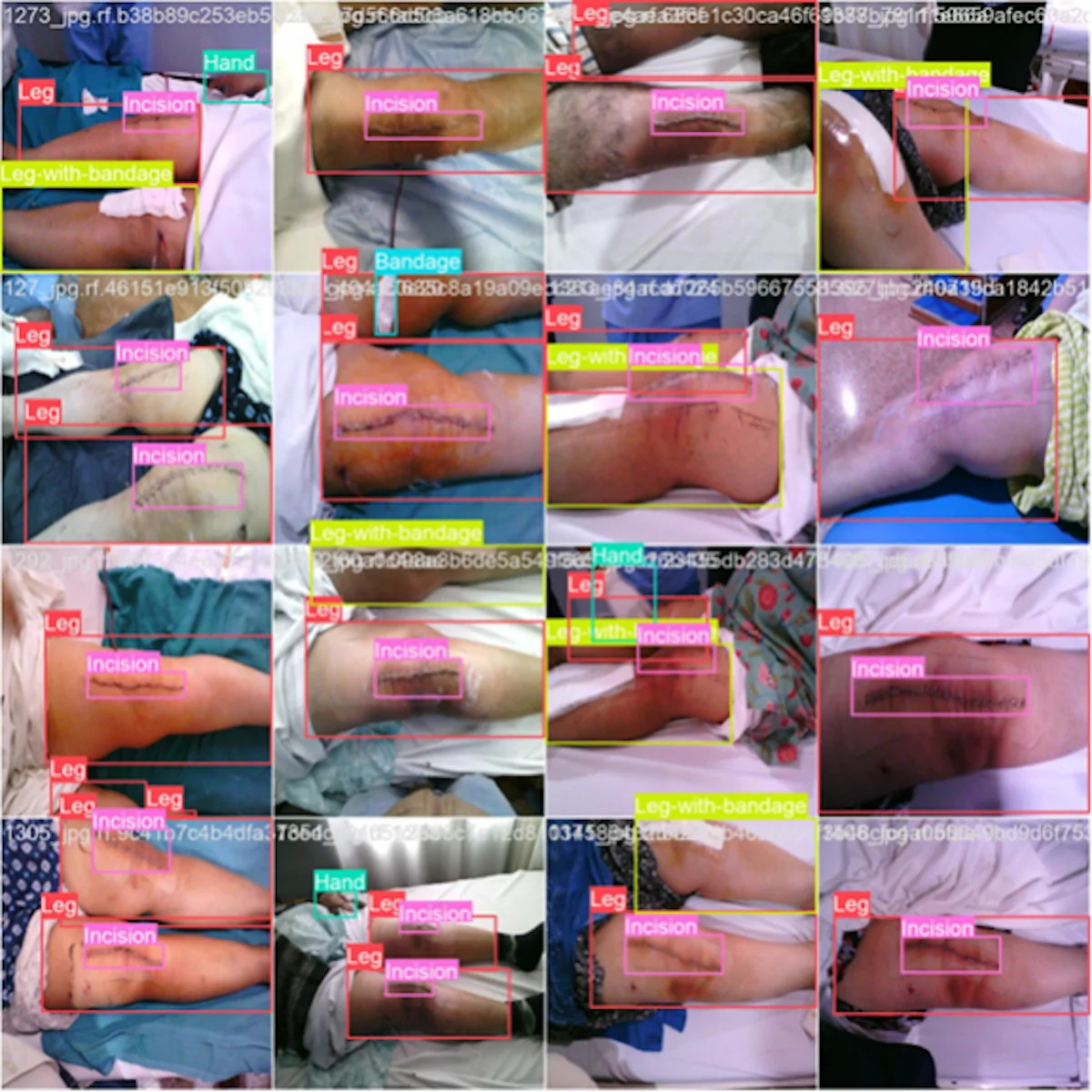
Representative anatomical landmark detections on held-out evaluation images. Predicted bounding boxes are shown for the surgical incision, operative limb, and exclusion regions at the fixed operating confidence of 0.5.

#### Wound Segmentation Model

The wound-tissue segmentation model delineated the 4 tissue classes at a fixed threshold of 0.5 and native image resolution. Micro-averaged across classes, the model achieved a sensitivity of 0.845 (95% CI 0.833-0.857), a positive predictive value of 0.759 (95% CI 0.744-0.772), and a dice coefficient of 0.800 (95% CI 0.790-0.808); the corresponding macro-averaged values were a sensitivity of 0.828 (95% CI 0.802-0.850), a positive predictive value of 0.740 (95% CI 0.722-0.758), and a dice coefficient of 0.778 (95% CI 0.758-0.795).

Because tissue pixels represented only 4.4% of all pixels, discrimination is most informatively summarized by the area under the precision-recall curve, which was 0.881 (95% CI 0.870-0.890) micro-averaged, against a chance value of 0.044, an approximately 20-fold improvement over chance. Predicted probabilities were well calibrated (Brier score 0.014; expected calibration error 0.010). Figure 5 illustrates a representative example of IAE correctly delineated on postoperative imaging. Aggregate segmentation metrics are reported in full in Multimedia Appendix 2.

**Figure 5:**
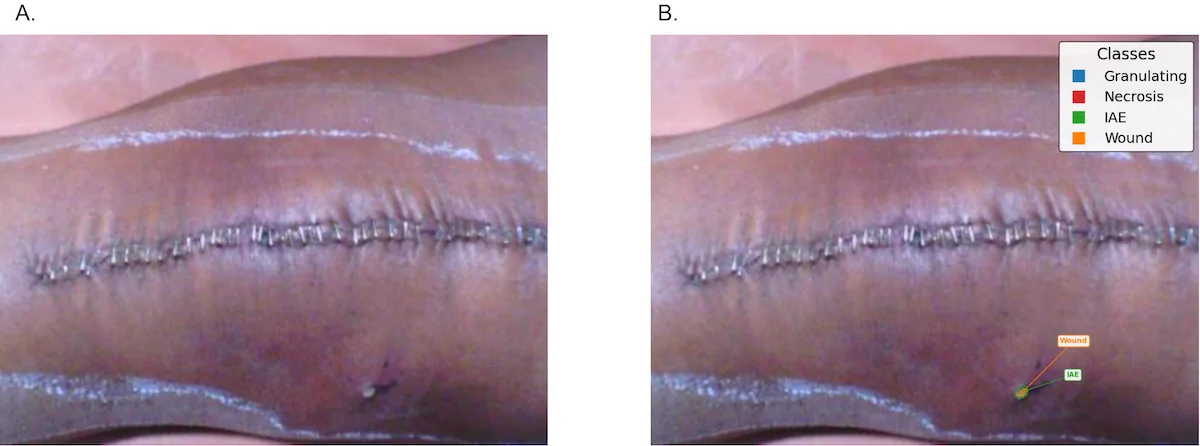
Wound-tissue segmentation output on a patient with active infection-associated exudate. (A) Electro-optical image (original); (B) Predicted multilabel tissue mask (purulent discharge/infection-associated exudate and open-wound field) is overlaid on the electro-optical image, illustrating pixel-level delineation of the infection-affected area used for tracking and remote monitoring. Prediction at a fixed threshold of 0.5 and native image resolution. IAE: infection-associated exudate.

#### Contextual Visual Reasoning

A VLM was incorporated into the architecture to provide protocol-specific cues and to disambiguate visually confusing findings that challenge conventional image classifiers. Blood at the incision is the representative case: its red appearance can be confused with red-colored background objects by conventional classification models, whereas a VLM can draw on scene context to resolve such ambiguity. Detection of blood presence/hematologic event was therefore the core reasoning task for the VLM, with infection classification examined on an exploratory basis. Both are image-level classification targets within the model-development corpus and are distinct from the clinician-adjudicated infection-related outcome of the study cohort. The model was Google’s Gemini 2.5 Flash, accessed and fine-tuned through Vertex AI.

Fine-tuning produced consistent improvements for both classes. The training data included 1052 blood presence and 1379 infection annotations across 17712 images, and the independent validation set of 1956 images contained 132 and 152 positive annotations for these classes, respectively.

For blood presence, validation accuracy increased from 96.3% with the base model to 96.7%, and the *F*_1_-score improved from 70.0% to 75.8%, driven primarily by an increase in recall from 63.6% to 75.8% (a 19.0% relative improvement) with only a modest reduction in precision from 77.8% to 75.8%. The fine-tuned model identified 16 additional positive cases, reducing false negatives from 48 to 32. On exploratory evaluation of the infection class, larger relative gains were observed: validation accuracy increased from 67.9% to 91.4%, and the *F*_1_-score rose from 24.9% to 55.3% (a 122.3% relative improvement). Infection precision increased from 15.2% to 46.4% (205.4% relative improvement), while recall remained unchanged at 68.4%, as fine-tuning reduced false-positive infection predictions from 580 to 120, a 79.3% reduction without increasing the number of missed cases. Expressed as clinical operating characteristics, the fine-tuned model achieved, for blood presence, a sensitivity of 75.8%, specificity of 98.2%, positive predictive value of 75.8%, and negative predictive value of 98.2%; and, for infection, a sensitivity of 68.4%, specificity of 93.3%, positive predictive value of 46.4%, and negative predictive value of 97.2% (full matrices and 95% CIs are provided in Multimedia Appendix 2). Because both classes are infrequent (approximately 7% prevalence), the negative predictive values are correspondingly high.

Overall, these results support the VLM’s core role in resolving visually confusing findings such as blood presence and indicate that domain-specific fine-tuning is essential for this task: the general-purpose base model performed less well, while fine-tuning improved blood presence classification and a markedly improved, though still exploratory, infection classifier.

#### Infrared Alignment, ROI Extraction, and Radiometric Inference

EO detections were transferred to thermal space by the deterministic EO-to-infrared projection, restricting radiometric analysis to the surgical field (Figure 6).

Within the projected region, the infrared region-of-interest extraction model isolated the radiometric field (Figure 7) on a patient-disjoint held-out set of 403 images from 178 patients, with a mean intersection-over-union of 0.985 (95% CI 0.984-0.986) and a Dice coefficient of 0.993; the median image-level intersection-over-union was 0.987, and no image fell below 0.90. The decision threshold was fixed on the training set before evaluation.

The ROI was passed as input to the radiometric classifier, which discriminated infection-related complications with an AUROC of 0.978 (95% CI 0.959-0.992) under out-of-fold, patient-grouped cross-validation. At a representative operating point, sensitivity was 0.808 (95% CI 0.65-0.92), specificity 0.974 (95% CI 0.96-0.99), positive predictive value 0.618 (95% CI 0.49-0.78), and negative predictive value 0.990 (95% CI 0.98-1.00); higher-sensitivity thresholds provided a rule-out configuration with a negative predictive value near 0.99, and higher-specificity thresholds a rule-in configuration. Complete per-threshold operating characteristics, indexed by the number needed to evaluate, are provided in Multimedia Appendix 2.

**Figure 6:**
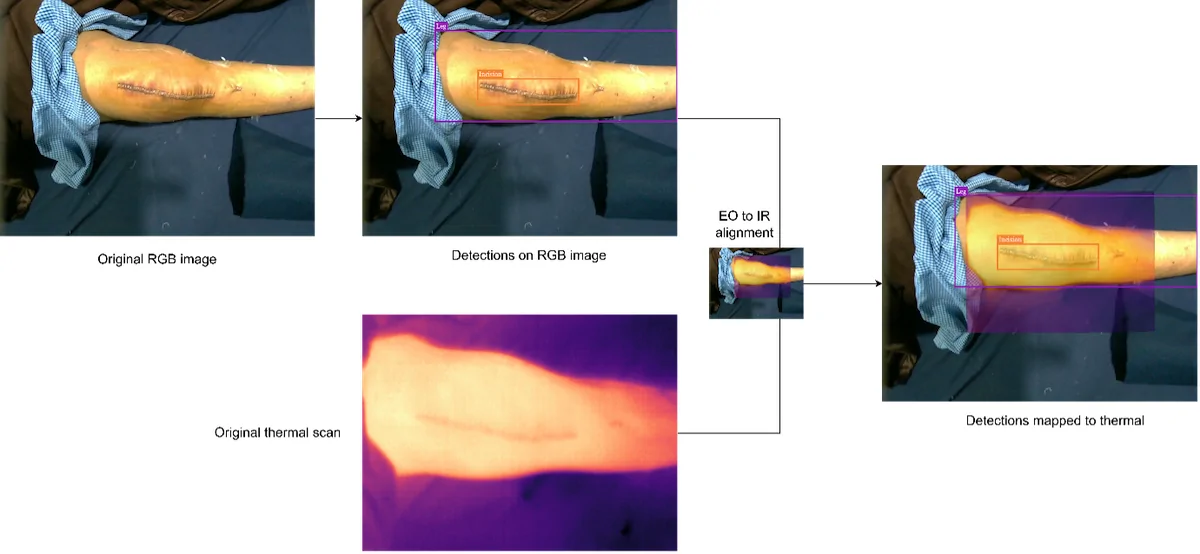
Electro-optical-to-infrared projection. Landmark detections established in the electro-optical frame are transferred to co-registered thermal space by a deterministic geometric mapping that preserves geometry and restricts radiometric analysis to the surgical field while suppressing environmental background. Panels show the EO detection (left) and the resulting projected region on the radiometric thermal frame (right). EO: electro-optical; RGB: red, green, and blue.

**Figure 7:**
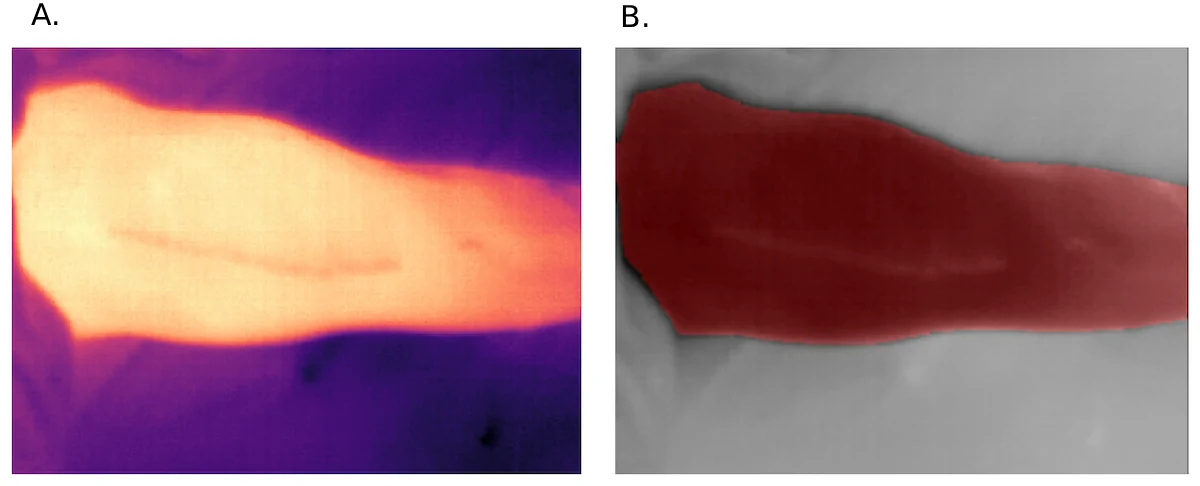
Thermal region-of-interest extraction on held-out evaluation images. (A) Thermal image (original); (B) Segmentation mask overlay (red).

### Clinical Risk Stratification

Age and gender were not associated with infection-related complications after multiplicity correction (both q>.5), and no baseline laboratory variable remained significantly associated after correction. Complication rates did not differ by surgical approach (robotic-assisted: 12/383, 3%; conventional: 14/366, 4%; Fisher exact: OR 0.81; *P*=.69). In unadjusted analyses, chronic kidney disease alone (OR 8.52, 95% CI 1.68-43.21) and chronic kidney disease and diabetes mellitus (OR 14.98, 95% CI 2.61-85.81) produced the largest associations with infection-related complications; neither persisted after Benjamini-Hochberg or Holm-Bonferroni correction. These associations are hypothesis-generating and warrant evaluation in an adequately powered cohort. Baseline laboratory and comorbidity cross-associations are reported in Multimedia Appendix 1.

## Discussion

### Principal Findings

To our knowledge, this is the first study to evaluate the technical feasibility of a multimodal AI pipeline integrating EO and infrared imaging for postoperative wound assessment after TKA. Each stage of the pipeline performed to a level sufficient to support the next. The anatomical landmark detector established a reliable surgical coordinate frame, the segmentation model characterized wound-tissue states, and infrared region-of-interest extraction isolated the radiometric field with near-ceiling accuracy on a patient-disjoint set. At the clinical end point, the radiometric classifier discriminated infection-related complications with an AUROC of 0.978 (95% CI 0.959-0.992) under out-of-fold, patient-grouped cross-validation. The classifier was framed as a combined detection and prediction task, in which every scan of a complication-affected knee contributed to the positive class; under this framing, it recovered a thermal signal in scans acquired before clinical recognition, and discrimination restricted to established complications was comparable (AUROC 0.981), indicating that the inclusion of pre–onset scans did not inflate performance.

The operating characteristics support 2 deployment modes. Configured for rule-out, the classifier achieved a negative predictive value near 0.99, appropriate to a surveillance workflow in which a negative result provides reassurance and a positive result prompts clinical review. Configured for rule-in, positive predictive value increased at the cost of sensitivity, suitable for prioritizing knees for escalation. Acquisition was performed in both clinic and home settings, indicating that the pipeline tolerates real-world variability. Exploratory analyses of baseline clinical and laboratory factors identified no association with infection-related complications that survived correction for multiple comparisons.

### Comparison to Prior Work

Reported experience with postarthroplasty thermography has been limited in scale; a recent meta-analysis pooled 318 patients across 10 studies [26]. To our knowledge, the present cohort is the largest to which 2D thermography with AI-based analysis has been applied for postarthroplasty surveillance. A methodological contrast with larger studies of postoperative skin temperature is instructive. Sharma et al [27] used single-point infrared thermometry, which yields on the order of tens of thousands of measurements per patient. The present system acquires 240×320 radiometric thermal frames co-registered with 640×480 EO data, on the order of tens of thousands of thermal measurements per acquisition and approximately 1.5 million per patient across the follow-up schedule. This difference of roughly 4 orders of magnitude is what permits AI-based segmentation, region-of-interest restriction, and spatial thermal analysis, which point thermometry cannot support.

Relative to established noninvasive adjuncts for PJI, the observed discrimination compares favorably. Serum C-reactive protein and erythrocyte sedimentation rate are widely used screening tests whose reported discrimination is generally at or below that observed here, and both require venipuncture. The present measurement is noncontact and can be repeated at each clinic or home visit. Prior imaging work has generally addressed a single modality. Muaddi et al [11] reported an AUC of 0.81 for infection classification from EO imaging alone, with high accuracy for incision detection comparable to the landmark performance reported here; the present pipeline adds wound-tissue segmentation and infrared integration. A multimodal study combining patient-reported symptoms with wound photographs reported an AUC of 0.76 for confirmed surgical site infection [12]. Together these comparisons indicate that combining structural EO analysis with radiometric infrared data provides information beyond that available from wound photography alone.

### Clinical Implications

Early intervention materially changes outcomes in PJI. Debridement, antibiotics, and implant retention succeed in approximately 93% of cases treated within one week of symptom onset, and pooled failure rates rise from 34.2% in acute early infection to 73.6% in late chronic infection [13,14]. A noncontact assessment that can be repeated at every clinic or home visit and that reliably clears knees not requiring escalation could support more consistent surveillance during the period when intervention is most effective. Given the projected economic burden of knee PJI in the United States [2], even modest improvements in early detection could yield meaningful savings, although formal cost-effectiveness analysis remains necessary. The exploratory finding that chronic kidney disease and diabetes mellitus carried the largest unadjusted associations with complications, though none survived multiplicity correction, is hypothesis-generating and could inform enrichment of higher-risk groups in future studies. Whether any of these operating characteristics translate into earlier intervention and improved outcomes can only be established in a prospective comparative study.

### Limitations

Several limitations should be considered. The study was conducted at a single center, and all reported performance estimates derive from held-out evaluation within that cohort; external validation in independent institutions and populations has not been performed and is required to establish generalizability. The cohort of 749 patients included only 26 complication cases; this small number of positive events limits power for rare outcomes, quantizes sensitivity in steps of approximately 0.04, and widens the CIs around performance and risk-factor estimates, so point estimates should be interpreted as feasibility signals rather than definitive performance. Mean ambient temperature differed between groups before clinical onset, indicating a difference in acquisition setting; its effect was quantified and bounded through an ambient-invariant representation. The follow-up schedule was designed to capture early postoperative complications and may have missed delayed presentations. Outcome adjudication relied on clinician judgment rather than standardized diagnostic criteria, which reflects real-world practice but may introduce variability. The vision-language component was evaluated as an auxiliary image-level classifier with modest precision for the infection class and did not contribute to the primary outcome. Certain algorithmic components are proprietary; to support independent assessment, the validation strategy and the controls for data leakage and confounding are described in detail, although the underlying model specifications are not disclosed. Finally, systematic evaluation of performance across skin tones, body habitus, surgical techniques, and socioeconomic groups was not undertaken in this feasibility study; such evaluation is a prerequisite for equitable clinical deployment and should be prioritized in future multicenter validation studies.

### Conclusions

A multimodal EO and infrared imaging pipeline was technically feasible for postoperative wound assessment after TKA in a large single-center cohort, achieving discrimination for infection-related complications that compared favorably with established noninvasive adjuncts while requiring neither contact nor venipuncture and tolerating both clinic and in-home acquisition. These findings establish proof of concept and define the technical requirements for validation. Prospective multicenter studies with external validation and randomized trials comparing AI-assisted monitoring with standard care on outcomes such as time to detection, complication severity at diagnosis, salvage success, and cost-effectiveness are the necessary next steps. Extending coverage to additional complications, including deep vein thrombosis and implant-related failure, is a further direction. Infrared-based vascular assessment has already shown promise, with a reported sensitivity of 96.9% for deep vein thrombosis [21]. Integrating patient-reported outcomes, wearable-derived data, and laboratory trajectories into a multimodal framework could enable more holistic physiologic modeling and support precision postoperative surveillance.

## Appendix

Multimedia Appendix 1 Exploratory Statistics.

Multimedia Appendix 2 Model Development, Validation Controls, and Detailed Performance.

## Abbreviations

AUC: area under the curve
AUROC: area under the receiver operating characteristic curve
COCO: Common Objects in Context
EO: electro-optical
GenAI: generative AI
IAE: infection-associated exudate
NICE: National Institute for Health and Care Excellence
OR: odds ratio
PJI: periprosthetic joint infection
ROI: region of interest
TKA: total knee arthroplasty
TRIPOD: Transparent Reporting of a multivariable prediction model for Individual Prognosis Or Diagnosis
VLM: vision-language model
WHO: World Health Organization

## Data Availability

The datasets generated and analyzed during this study are not publicly available due to proprietary restrictions related to ongoing algorithm development and intellectual property protection. Deidentified data supporting the findings of this study may be made available from the corresponding author upon reasonable request, subject to data use agreements and sponsor approval.
