# Supplementary material for: Technical Feasibility of a Multimodal Electro-Optical and Infrared Imaging Pipeline for Postoperative Wound Assessment After Total Knee Arthroplasty: Formative Evaluation Study
Source: JMIR Form Res. 2026 Oct 8;10:e99244. doi: 10.2196/99244 (PMC13691468; doi:10.2196/99244)
Supplement: Supplementary file 1 [file formative_v10i1e99244_app1.docx]

# Multimedia Appendix 1. Baseline Characteristics and Exploratory Statistical Analyses

Manuscript #99244: Technical Feasibility of a Multimodal Electro-Optical and Infrared Imaging Pipeline for Postoperative Wound Assessment After Total Knee Arthroplasty: A Formative Evaluation Study

This appendix reports the baseline characteristics of the study cohort (Tables 1 - 4) and the exploratory analyses of associations among baseline demographic, laboratory, and comorbidity variables. The exploratory analyses (Tables 5-10) do not bear directly on the study aims and are reported here in full for completeness. All analyses were performed on the full study cohort (N=749).

**Statistical software.** Python 3.12.7 with NumPy 2.1.2, pandas 2.2.3, SciPy 1.14.1, statsmodels 0.14.4, and Matplotlib 3.9.2.

**Methods.** All tests were two-tailed with α=.05. Two-group comparisons of continuous variables used the Mann–Whitney U test with Cliff delta as the effect size, or the Student’s *t* test with Hedges *g* where the normality assumption was satisfied. Multi-group comparisons used the Kruskal-Wallis test with epsilon squared. Continuous–continuous associations used the Spearman rank correlation. Categorical associations used the chi-square test with Cramér *V*, or the Fisher exact test where any expected cell count was below 5. Binary exposure–outcome relationships are expressed as odds ratios with 95% confidence intervals, with the Haldane–Anscombe correction applied only where a cell count was zero. Multiplicity was controlled using the Benjamini-Hochberg false discovery rate procedure (*q*) as the primary correction, with Holm–Bonferroni reported for strong family-wise error control. Confidence intervals for proportions are Wilson intervals.

## Baseline characteristics of the study cohort

Demographic, procedural, laboratory, and comorbidity characteristics of the 749 patients comprising the analytic cohort (Tables A.1.1 - 4). Continuous variables are reported as mean (SD) with 95% CI and as median (IQR); normality was assessed by the Shapiro–Wilk test. Proportions are reported as numerator/denominator with Wilson 95% CI.

### Table A.1.1. Demographics and procedure

| **Variable** | **Value** | **95% CI** |
| --- | --- | --- |
| Age, years — mean (SD) | 63.4 (9.1) | 62.7–64.0 |
| Age, years — median (IQR) | 64 (57–70) | — |
| Age, years — range | 30–90 | — |
| Female | 525/749 (70.1%) | 66.7–73.3 |
| Male | 224/749 (29.9%) | 26.7–33.3 |
| Robotic-assisted | 383/749 (51.1%) | 47.6–54.7 |
| Conventional | 366/749 (48.9%) | 45.3–52.4 |
| Laterality: right | 327/749 (43.7%) | 40.1–47.2 |
| Laterality: left | 292/749 (39.0%) | 35.6–42.5 |
| Laterality: bilateral | 130/749 (17.4%) | 14.8–20.2 |

### Table A.1.2. Baseline laboratory values (n=749)

| **Variable** | **Mean (SD)** | **95% CI** | **Median (IQR)** | **Range** |
| --- | --- | --- | --- | --- |
| Hemoglobin, g/dL | 12.29 (1.50) | 12.18–12.40 | 12.3 (11.2–13.3) | 7.9–18.8 |
| Creatinine, mg/dL | 0.87 (0.23) | 0.86–0.89 | 0.83 (0.71–0.99) | 0.50–2.73 |
| Sodium, mmol/L | 137.8 (3.7) | 137.6–138.1 | 138 (136–140) | 100.5–148 |
| Potassium, mmol/L | 4.36 (0.56) | 4.32–4.40 | 4.3 (4.1–4.6) | 2.9–9.5 |

Haemoglobin was consistent with a normal distribution (Shapiro–Wilk *P*=.23); age, creatinine, sodium, and potassium were not (all *P*<.01).

### Table A.1.3. Comorbidities

| **Comorbidity** | **n/N (%)** | **95% CI** |
| --- | --- | --- |
| Hypertension | 521/749 (69.6%) | 66.2–72.7 |
| Diabetes mellitus | 306/749 (40.9%) | 37.4–44.4 |
| Thyroid disease | 168/749 (22.4%) | 19.6–25.6 |
| Coronary artery disease | 69/749 (9%) | 7.3–11.5 |
| Asthma | 39/749 (5%) | 3.8–7.0 |
| Cerebrovascular accident | 21/749 (3%) | 1.8–4.2 |
| Chronic kidney disease | 9/749 (1%) | 0.6–2.3 |
| Epilepsy | 7/749 (1%) | 0.5–1.9 |
| Rheumatoid arthritis | 7/749 (1%) | 0.5–1.9 |
| Parkinson disease | 4/749 (<1%) | 0.2–1.4 |
| Obstructive sleep apnoea | 2/749 (<1%) | 0.1–1.0 |
| Urinary tract infection | 2/749 (<1%) | 0.1–1.0 |
| Bronchitis, coronary angiography, interstitial lung disease, psoriasis | 1/749 each (<1%) | 0.02–0.8 |

### Table A.1.4. Primary outcome

| **Outcome** | **n/N (%)** | **95% CI** |
| --- | --- | --- |
| Infection-related complication | 26/749 (3%) | 2.4–5.0 |

## Age associations

### Table A.1.5. Age Associations (N=749). Associations significant after false discovery rate correction are listed. Older age was associated with hypertension, diabetes mellitus, and coronary artery disease, with higher creatinine and potassium and lower sodium, and differed by laterality, surgery type, and sex. These are expected demographic and physiological relationships and are not specific to the study aims.

| **Comparison** | **Test** | **Effect size** | **95% CI** | ***P*** | ***q* (FDR)** |
| --- | --- | --- | --- | --- | --- |
| Age × laterality | Kruskal–Wallis | ε²=0.130 | — | <.001 | <.001 |
| Creatinine × age | Spearman ρ | ρ=0.253 | 0.186 to 0.318 | <.001 | <.001 |
| Age × hypertension | Mann–Whitney U | Cliff δ=−0.302 | −0.392 to −0.212 | <.001 | <.001 |
| Age × surgery type | Kruskal–Wallis | ε²=0.038 | — | <.001 | <.001 |
| Age × sex | Kruskal–Wallis | ε²=0.037 | — | <.001 | <.001 |
| Sodium × age | Spearman ρ | ρ=−0.162 | −0.234 to −0.089 | <.001 | <.001 |
| Potassium × age | Spearman ρ | ρ=0.156 | 0.087 to 0.223 | <.001 | <.001 |
| Age × diabetes mellitus | Mann–Whitney U | Cliff δ=−0.169 | −0.248 to −0.086 | <.001 | <.001 |
| Age × coronary artery disease | Mann–Whitney U | Cliff δ=−0.276 | −0.399 to −0.150 | <.001 | <.001 |

## Sex associations

## Table A.1.6. Sex associations (N=749). Associations significant after false discovery rate correction are listed. Male patients had higher creatinine and hemoglobin, lower sodium, higher prevalence of coronary artery disease, and lower prevalence of thyroid disease. These reflect established sex-related physiological differences.

| **Comparison** | **Test** | **Effect size** | **95% CI** | ***P*** | ***q* (FDR)** |
| --- | --- | --- | --- | --- | --- |
| Sex × creatinine | Mann–Whitney U | Cliff δ=0.593 | 0.525 to 0.658 | <.001 | <.001 |
| Sex × haemoglobin | Mann–Whitney U | Cliff δ=0.499 | 0.413 to 0.581 | <.001 | <.001 |
| Sex × age | Mann–Whitney U | Cliff δ=0.247 | 0.156 to 0.337 | <.001 | <.001 |
| Sex × thyroid disease | Fisher exact | — | — | <.001 | <.001 |
| Sex × sodium | Mann–Whitney U | Cliff δ=−0.152 | −0.242 to −0.060 | <.001 | .005 |
| Sex × laterality | Chi-square | Cramér *V*=0.123 | — | .001 | .005 |
| Sex × coronary artery disease | Fisher exact | — | — | .01 | .04 |

## Laboratory–comorbidity associations

**Table A.1.7.** Laboratory–comorbidity associations (N=749). Associations significant after false discovery rate correction are listed. Creatinine was higher in patients with chronic kidney disease, hypertension, diabetes mellitus, and coronary artery disease. Diabetes mellitus showed a coherent profile of higher potassium with lower sodium and haemoglobin. Thyroid disease was associated with lower haemoglobin. The very large effect size for creatinine in chronic kidney disease (Cliff δ=0.960) reflects the laboratory basis of that diagnosis.

| **Comparison** | **Test** | **Effect size** | **95% CI** | ***P*** | ***q* (FDR)** |
| --- | --- | --- | --- | --- | --- |
| Creatinine × hypertension | Mann–Whitney U | Cliff δ=0.273 | 0.186 to 0.357 | <.001 | <.001 |
| Potassium × diabetes mellitus | Mann–Whitney U | Cliff δ=0.224 | 0.142 to 0.305 | <.001 | <.001 |
| Creatinine × chronic kidney disease | Mann–Whitney U | Cliff δ=0.960 | 0.907 to 0.992 | <.001 | <.001 |
| Sodium × diabetes mellitus | Mann–Whitney U | Cliff δ=−0.191 | −0.270 to −0.109 | <.001 | <.001 |
| Haemoglobin × diabetes mellitus | Mann–Whitney U | Cliff δ=−0.181 | −0.262 to −0.100 | <.001 | <.001 |
| Creatinine × diabetes mellitus | Mann–Whitney U | Cliff δ=0.175 | 0.091 to 0.258 | <.001 | <.001 |
| Haemoglobin × thyroid disease | Student *t* | Hedges *g*=−0.274 | −0.448 to −0.107 | .002 | .02 |
| Creatinine × coronary artery disease | Mann–Whitney U | Cliff δ=0.211 | 0.067 to 0.351 | .004 | .03 |

## Comorbidity associations with infection-related complication

Table A.1.8. Comorbidity associations with infection-related complication (N=749). No association reached significance after correction for multiple comparisons. Odds ratios are presented unadjusted.

| **Comorbidity** | **n with comorbidity** | **Odds ratio** | **95% CI** | ***P* (Fisher)** | ***q* (FDR)** |
| --- | --- | --- | --- | --- | --- |
| Chronic kidney disease | 9 | 8.52 | 1.68 to 43.21 | .04 | .24 |
| Cerebrovascular accident | 21 | 1.41 | 0.18 to 10.90 | .53 | .98 |
| Asthma | 39 | 1.55 | 0.35 to 6.79 | .64 | >.99 |
| Hypertension | 521 | 0.82 | 0.36 to 1.87 | .67 | >.99 |
| Coronary artery disease | 69 | 1.30 | 0.38 to 4.44 | .73 | >.99 |
| Thyroid disease | 168 | 0.82 | 0.30 to 2.20 | .81 | >.99 |
| Diabetes mellitus | 306 | 1.06 | 0.48 to 2.35 | >.99 | >.99 |
| Epilepsy | 7 | 1.80 | 0.10 to 32.40 | >.99 | >.99 |

## Baseline laboratory values and infection-related complication

## Table A.1.9. Baseline laboratory values and infection-related complication (N=749). No laboratory variable was associated with infection-related complication.

| **Variable** | **Test** | **Effect size** | **95% CI** | **OR per 1 SD** | **95% CI** | ***P*** | ***q* (FDR)** |
| --- | --- | --- | --- | --- | --- | --- | --- |
| Potassium | Mann–Whitney U | Cliff δ=−0.160 | −0.364 to 0.056 | 0.72 | 0.45 to 1.15 | .16 | .51 |
| Creatinine | Mann–Whitney U | Cliff δ=−0.104 | −0.327 to 0.139 | 0.86 | 0.54 to 1.38 | .37 | .71 |
| Hemoglobin | Student *t* | Hedges *g*=−0.135 | −0.600 to 0.265 | 0.87 | 0.57 to 1.34 | .50 | .77 |
| Sodium | Mann–Whitney U | Cliff δ=−0.035 | −0.248 to 0.163 | 0.84 | 0.62 to 1.14 | .76 | .90 |

## Comorbidity combinations and infection-related complication

**Table A.1.10.** Comorbidity combinations and infection-related complication (N=749). A scan of 143 comorbidity combinations was performed. No combination was significant after false discovery rate correction (all *q*>.99). Combinations with the largest point estimates are shown in Table A.1.10. Cell counts are small and confidence intervals correspondingly wide; these results are hypothesis-generating only.

| **Combination*** | **n with combination** | **Complications among them** | **Odds ratio** | **95% CI** | ***P* (Fisher)** | ***q* (FDR)** |
| --- | --- | --- | --- | --- | --- | --- |
| CKD + DM | 6 | 2 | 14.98 | 2.61 to 85.81 | .02 | >.99 |
| CAD + CKD + DM | 3 | 1 | 14.42 | 1.27 to 164.35 | .10 | >.99 |
| CAD + CKD | 4 | 1 | 9.60 | 0.96 to 95.57 | .13 | >.99 |
| CKD alone | 9 | 2 | 8.52 | 1.68 to 43.21 | .04 | >.99 |
| CKD + DM + HTN | 5 | 1 | 7.19 | 0.78 to 66.69 | .16 | >.99 |
| CKD + HTN | 7 | 1 | 4.78 | 0.55 to 41.21 | .22 | >.99 |

*CAD, coronary artery disease; CKD, chronic kidney disease; DM, diabetes mellitus; HTN, hypertension.

Every elevated point estimate in this table rests on one or two complication events. The pattern is consistent with chronic kidney disease, particularly in combination with diabetes mellitus, marking a higher-risk group, but the cohort contains too few events in these strata to support inference, and no association survived multiplicity correction.
