# Supplementary material for: Technical Feasibility of a Multimodal Electro-Optical and Infrared Imaging Pipeline for Postoperative Wound Assessment After Total Knee Arthroplasty: Formative Evaluation Study
Source: JMIR Form Res. 2026 Oct 8;10:e99244. doi: 10.2196/99244 (PMC13691468; doi:10.2196/99244)
Supplement: Supplementary file 2 [file formative_v10i1e99244_app2.docx]

# Multimedia Appendix 2. Model Development, Validation Controls, and Detailed Performance

Manuscript #99244: Technical Feasibility of a Multimodal Electro-Optical and Infrared Imaging Pipeline for Postoperative Wound Assessment After Total Knee Arthroplasty: A Formative Evaluation Study

## A2.1 Data sources

### Study cohort

The prospective cohort of 749 patients provided all clinical outcome data, the exploratory comorbidity and laboratory analyses (Multimedia Appendix 2), and all radiometric thermal data used for the infrared analyses.

### Publicly sourced datasets

Electro-optical model development combined institution-acquired images with publicly available images obtained from open image repositories (Table A.2.1). Publicly sourced datasets contributed anatomical classes (hand, foot, lower-body region, neck) and additional wound and tissue examples. All datasets used were distributed under the MIT License or the Creative Commons Attribution 4.0 International License.

**Table A.2.1. External datasets used in analyses.**

| Dataset | Purpose | License | Availability at time of writing |
| --- | --- | --- | --- |
| bodyparts-pql5q/tissues-v3b6t-7lu1r | Wound tissue | MIT / CC BY 4.0 | No longer available |
| bodyparts-pql5q/fitnessbodyregressiondetection-3-zcd87 | Anatomical regions | MIT / CC BY 4.0 | Available |
| bodyparts-pql5q/feet-detection-lc4xy-rkf80 | Foot detection | MIT / CC BY 4.0 | Available |
| bodyparts-pql5q/hand-test-obtm5-onlak | Hand detection | MIT / CC BY 4.0 | Available |
| segseg-qv5sk-nv62k | Wound segmentation | MIT / CC BY 4.0 | No longer available |
| wound_datasetb_apg | Wound segmentation | MIT / CC BY 4.0 | No longer available |
| bharanikumar/wound-tissue-classification | Wound tissue | MIT / CC BY 4.0 | No longer available |
| lamprosdionysiou/lowerbodyparts | Anatomical regions | MIT / CC BY 4.0 | Available |
| test-zsimf/lowerbodydetection-8hdaq | Anatomical regions | MIT / CC BY 4.0 | Available |
| florence-fine-tune/neck-laa8i | Anatomical region | MIT / CC BY 4.0 | No longer available |

All datasets were accessed from the Roboflow Universe repository (https://universe.roboflow.com). Several have since been withdrawn by their maintainers and are noted accordingly. Annotation of institution-acquired images was performed using the Roboflow annotation platform.

### Augmentation

Training data was expanded by morphological transformation and by composited edits in which complication features and confounding objects were introduced across varying scenarios, in order to increase representation of infrequent complication appearances. **Augmented and composited images were used for model training only and appear in no evaluation set. All evaluation sets comprise unaltered images.**

## A2.2 Thermal classifier - cohort and evaluation set

The thermal analyses were conducted on the radiometric image series contributed by the study cohort. The primary evaluation applied a postoperative day (POD) ≥14 window, yielding the 529 knees (26 complication, 503 control; prevalence 4.91%) reported in the main text; within this window there were 409 patients. Knees imaged only earlier in the postoperative course were retained for the secondary full-cohort analysis (A3.4). Every reported score is out-of-fold from five-fold cross-validation with folds grouped by person, averaged over three random seeds.

## A2.3 Validation controls applied to the thermal classifier

Each control is stated with the measurement demonstrating its effect.

### Out-of-fold evaluation

Every reported score is produced by a model fitted without the patient being scored. The cohort is partitioned into five folds; each model is fitted on four and predicts the fifth. This applies to all eight ensemble members, including the two logistic-regression members, which are refitted within the evaluation procedure rather than reused from any earlier fit. Fold disjointness is asserted programmatically at run time, and execution halts if any group appears on both sides of a split. Two knees from one person share body habitus, vasculature, imaging session, and room conditions. The measured within-person score correlation across bilateral pairs was *r*=0.44, which is why patient-level rather than knee-level grouping is required.

### No imputation of predicted scores

Scans acquired before clinical onset belong to complication-positive knees. Detection-labelled members exclude these scans from the training loss but still predict on them, and the resulting observed scores enter the knee-level aggregate. No missing value is filled with a summary statistic at any point in the pipeline. This matters because any fill value applied to pre-onset scans would affect only the positive class and would therefore shift class separation.

### No fitted combination layer

Members are combined by equal-weight rank averaging. The combination step contains no parameters fitted to labels and therefore cannot overfit the evaluation folds. A nested performance-weighted alternative was evaluated and produced 0.9666–0.9677 against 0.9664 for equal weights, confirming that the parameter-free combination costs no discriminative performance.

### Threshold selection

Single-figure operating points use nested threshold selection: a second grouped five-fold split over the knee-level scores, with the threshold chosen on training patients, applied to held-out patients, and decided by majority vote across folds and seeds. Operating characteristics across the full range of decision thresholds are tabulated in A3.4, indexed by number needed to evaluate, to support selection of a clinical threshold.

### Reference curves fitted on training controls only

The physics member expresses each feature as a deviation from expected uncomplicated healing at the same postoperative day. These reference curves are fitted on control scans from the training folds only and then applied to held-out scans, so that no held-out patient and no complication-positive case contributes to the definition of normal.

### Postoperative day excluded as a model input

Postoperative day was evaluated as a candidate input and excluded. Although label-free, its distribution differed between groups as a function of imaging schedule rather than pathology (Mann–Whitney *P*<.001; Kolmogorov–Smirnov *P*=.004). The classifier therefore uses thermal features and within-knee change-from-baseline metrics only, with postoperative-day-normative referencing rather than postoperative day itself as an input.

### Imaging distance

Texture and gradient statistics are computed per pixel, so the apparent size of the knee in frame can influence their values. Each scan is cropped to the region-of-interest bounding box with an 8% margin before resizing, holding pixel scale constant across scans. After this control, the correlation between knee-level score and region-of-interest area fraction among control patients was −0.19.

### Ambient temperature

Absolute thermal readings depend on the temperature of the room in which they were acquired. Ambient temperature was estimated per scan as the median of the region outside the region of interest. Mean ambient temperature differed between groups (27.35 °C for complication scans against 29.98 °C for control scans). This difference is present in scans acquired before clinical onset and is therefore a property of the acquisition setting rather than a consequence of outcome.

Two controls were applied. First, a self-normalised, ambient-invariant encoding references every channel to the knee’s own median and interquartile range, so that a uniform temperature shift of the scene cancels exactly; two of the eight members use this encoding, and as a standalone classifier it reaches an AUROC of 0.950. Second, per-member dependence on ambient temperature was measured among control patients and ranged from −0.04 to −0.17 across the convolutional members.

### Follow-up intensity

If patients who develop complications were imaged more frequently, scan count could act as a predictor. In the primary evaluation set, scan count alone gives an AUROC of 0.466, indicating no association. Knee-level aggregation uses the mean over scans, which does not scale with the number of scans available; maximum aggregation, which would reward additional scans, was evaluated and performed less well (0.956 against 0.966).

### Anatomical orientation

Augmentation is restricted to horizontal flips, which map between left and right knees, together with small translation and scale jitter. Imposing rotation invariance on anatomically oriented images degraded knee-level AUROC from 0.967 to 0.927. Canonical-frame regional features were computed over a 3×3 grid; all localization contrasts were at chance (focality 0.516, center-versus-periphery 0.520, hot-cell count 0.515), so no reported result depends on lesion position.

## A2.4 Detailed performance - thermal classifier

Primary evaluation set: postoperative day ≥14, n=529 knees (26 complication, 503 control), prevalence 4.91%. All scores out-of-fold, folds grouped by person, averaged over three seeds. Confidence intervals are 95% percentile intervals from patient-stratified bootstrap resampling (2,000 resamples). NNE denotes number needed to evaluate (1/positive predictive value).

### Ensemble of eight members — AUROC 0.978 (95% CI 0.959–0.992)

Operating characteristics across the range of decision thresholds on the primary evaluation set, indexed by NNE (Table A.2.2). The representative operating point reported in the main text corresponds to NNE ≈ 1.6 (sensitivity 0.808, specificity 0.974, positive predictive value 0.618, negative predictive value 0.990). Higher-NNE thresholds give a high-sensitivity, high-NPV rule-out configuration; lower-NNE thresholds give a high-PPV rule-in configuration. Individual member performance across different thermal encodings and labeling schemes is detailed in Table A.2.3.

**Table A.2.2. Operating characteristics by NNE**

| **NNE** | **Sensitivity** | **Specificity** | **PPV** | **NPV** | **Accuracy** |
| --- | --- | --- | --- | --- | --- |
| 1.2 | 0.577 (0.38–0.77) | 0.994 (0.99–1.00) | 0.833 (0.68–1.00) | 0.978 (0.97–0.99) | 0.974 |
| 1.4 | 0.577 (0.38–0.77) | 0.988 (0.98–1.00) | 0.714 (0.55–0.89) | 0.978 (0.97–0.99) | 0.968 |
| 1.6 | 0.808 (0.65–0.92) | 0.974 (0.96–0.99) | 0.618 (0.49–0.78) | 0.990 (0.98–1.00) | 0.966 |
| 1.8 | 0.808 (0.65–0.92) | 0.966 (0.95–0.98) | 0.553 (0.44–0.69) | 0.990 (0.98–1.00) | 0.958 |
| 2.0 | 0.846 (0.69–0.96) | 0.956 (0.94–0.97) | 0.500 (0.40–0.62) | 0.992 (0.98–1.00) | 0.951 |
| 2.5 | 0.846 (0.69–0.96) | 0.934 (0.91–0.95) | 0.400 (0.32–0.50) | 0.992 (0.98–1.00) | 0.930 |
| 2.8 | 0.885 (0.73–1.00) | 0.918 (0.89–0.94) | 0.359 (0.29–0.44) | 0.994 (0.99–1.00) | 0.917 |

### Table A.2.3. Ensemble member performance

| **Member** | **Encoding** | **Labelling** | **AUROC** |
| --- | --- | --- | --- |
| 1 | Physics | Detection | 0.969 |
| 2 | Colormap | Detection | 0.966 |
| 3 | Physics | Combined | 0.966 |
| 4 | Self-normalised | Detection | 0.948 |
| 5 | Colormap | Combined | 0.942 |
| 6 | Self-normalised | Combined | 0.937 |
| 7 | Self-supervised embedding + logistic regression | — | 0.889 |
| 8 | Physics features + normative scoring | — | 0.815 |

### Precision of sensitivity estimates

The primary evaluation set contains 26 complication-positive knees. Sensitivity is therefore quantised in steps of 1/26 (approximately 0.038), and its 95% confidence interval spans approximately ±0.18. Differences in sensitivity smaller than about 0.15 are not resolvable in this cohort. Positive predictive value is bounded by prevalence and is reported with its prevalence throughout.

## A2.5 Detailed performance - wound-tissue segmentation

Held-out evaluation set: 2,003 images. Multi-label, each class an independent binary task with background excluded; threshold 0.5, native image resolution. Positive-pixel prevalence 4.4%; chance PR-AUC is therefore 0.044. Confidence intervals from 1,000-image bootstrap. Detailed performance is shown in Table A.2.4.

**Table A.2.4. Segmentation metrics.**

| Metric | Macro (95% CI) | Micro (95% CI) |
| --- | --- | --- |
| Dice | 0.778 (0.758–0.795) | 0.800 (0.790–0.808) |
| Intersection-over-Union | 0.646 (0.621–0.668) | 0.666 (0.653–0.678) |
| Positive predictive value | 0.740 (0.722–0.758) | 0.759 (0.744–0.772) |
| Negative predictive value | 0.992 (0.991–0.993) | 0.993 (0.992–0.994) |
| Sensitivity | 0.828 (0.802–0.850) | 0.845 (0.833–0.857) |
| Specificity | 0.987 (0.986–0.988) | 0.988 (0.987–0.988) |
| Accuracy | 0.981 (0.980–0.982) | 0.981 (0.980–0.982) |
| Balanced accuracy | 0.907 (0.894–0.918) | 0.917 (0.910–0.922) |
| Matthews correlation coefficient | 0.770 (0.751–0.787) | 0.791 (0.781–0.800) |
| Cohen κ | 0.767 (0.747–0.784) | 0.790 (0.780–0.799) |
| AUROC | 0.970 (0.959–0.978) | 0.989 (0.986–0.991) |
| PR-AUC | 0.832 (0.803–0.858) | 0.881 (0.870–0.890) |
| Brier score | 0.014 | 0.014 |
| Expected calibration error | 0.010 | 0.010 |

A PR-AUC of 0.881 against a chance value of 0.044 represents approximately twentyfold discrimination above chance.

**Architecture.** TransNeXt-Tiny encoder (ImageNet-pretrained) with a UPerNet decode head and auxiliary deep supervision, trained at 640×640 in bf16 with exponential moving average and augmentation. A wound-gating head constrains tissue predictions to lie within the predicted wound region. Best checkpoint selected by validation macro-Dice.

## A2.6 Detailed performance - anatomical landmark detection

Evaluation set: 426 images, 914 annotated objects, 7 classes. Greedy matching at Intersection-over-Union ≥0.5; mean average precision under the COCO protocol. Operating-point metrics at a fixed, untuned confidence threshold of 0.5. Confidence intervals from 1,000-resample bootstrap over images. Detailed performance is shown in Table A.2.5.

**Table A.2.5. Detailed performance for landmark detection**

| Metric | Value, % (95% CI) |
| --- | --- |
| Mean average precision @[.5:.95] (primary) | 69.2 (65.5–72.6) |
| Mean average precision @0.5 | 88.6 (83.4–93.8) |
| Mean average precision @0.75 | 78.3 (73.4–83.3) |
| Mean Intersection-over-Union (matched detections) | 87.3 (86.4–88.3) |
| Sensitivity @0.5 | 96.7 (95.4–97.9) |
| Specificity @0.5 | 98.6 (98.1–99.0) |
| Positive predictive value @0.5 | 95.5 (94.0–97.0) |
| Negative predictive value @0.5 | 99.0 (98.5–99.4) |
| Accuracy @0.5 | 98.2 (97.6–98.7) |
| F1 @0.5 | 96.1 (95.0–97.2) |
| Matthews correlation coefficient @0.5 | 0.949 (0.933–0.963) |
| Macro / micro AUROC | 99.5 / 99.7 |

Perceptual-hash screening of all 8,635 training images against all 426 evaluation images identified no evaluation image within a near-duplicate Hamming distance (≤5) of any training image (median nearest-neighbour distance 14), supporting the absence of near-duplicate leakage between partitions.

## A2.7 Detailed performance - thermal region-of-interest extraction

Patient-disjoint evaluation set: 403 images from 178 held-out patients, with no patient or capture-session overlap with training data. Detailed performance is shown in Table A.2.6.

**Table A.2.6. Detailed performance for ROI extraction**

| Metric | Value |
| --- | --- |
| Mean Intersection-over-Union (threshold fixed on training data) | 0.985 |
| Mean Intersection-over-Union (default untuned threshold) | 0.985 |
| Dice | 0.993 |
| Pixel-level AUROC | 0.9997 |
| Median image Intersection-over-Union | 0.987 |
| Images below 0.90 Intersection-over-Union | 0 of 403 |

**Architecture.** U-Net decoder with EfficientNet-B4 encoder (ImageNet-pretrained), trained at 512×384 with a combined Dice, binary cross-entropy, and Lovász objective. Inference uses multi-scale (0.75, 1.0, 1.25) and horizontal-flip test-time augmentation, with probabilities reduced to native 320×240 resolution. The decision threshold (0.56) was selected on the training set and fixed before evaluation.

The task is binary knee-versus-background region isolation, for which near-ceiling performance is expected. Residual error is concentrated in boundary pixels at the frame edge where the knee is partially cut off or the annotation polygon is imprecise. Training images were lossless PNG and evaluation images were JPEG, introducing a minor encoding-domain difference between partitions.

## A2.8 Detailed performance - contextual vision–language model

The vision–language model was fine-tuned on a multi-label corpus of electro-optical wound images and evaluated on an independent, unaltered held-out set of 1,956 surgical-site images. Blood presence was the core reasoning task; infection classification was examined on an exploratory basis. Both are image-level classification targets within the model-development corpus and are distinct from the clinician-adjudicated infection-related outcome of the study cohort. Detailed performance is shown in Table A.2.7.

### Table A.2.7. VLM class composition

| Class | Training annotations (within 17,712 images) | Validation annotations (within 1,956 images) |
| --- | --- | --- |
| Infection | 1,379 | 152 |
| Blood presence | 1,052 | 132 |
| Purulent discharge | 728 | 68 |
| Open wound | 704 | 60 |
| Discharge or exudate | 516 | 56 |
| Cellulitis | 283 | 44 |

### Table A.2.8. Base versus fine-tuned summary

| Class | Metric | Base | Fine-tuned |
| --- | --- | --- | --- |
| Blood presence | Accuracy | 96.3% | 96.7% |
|  | F1 | 0.700 | 0.758 |
|  | Precision (PPV) | 0.778 | 0.758 |
|  | Recall (sensitivity) | 0.636 | 0.758 |
| Infection | Accuracy | 67.9% | 91.4% |
|  | F1 | 0.249 | 0.553 |
|  | Precision (PPV) | 0.152 | 0.464 |
|  | Recall (sensitivity) | 0.684 | 0.684 |

Fine-tuning improved blood-presence recall from 0.636 to 0.758 at essentially unchanged precision. For the infection class, fine-tuning raised precision from 0.152 to 0.464 with recall unchanged at 0.684, improving F1 from 0.249 to 0.553 (Table A.2.8).

### Table A.2.9. Clinical operating characteristics (fine-tuned model, Wilson 95% CI)

| Class | Sensitivity | Specificity | PPV | NPV | Accuracy |
| --- | --- | --- | --- | --- | --- |
| Blood presence | 0.758 (0.678–0.823) | 0.982 (0.975–0.988) | 0.758 (0.678–0.823) | 0.982 (0.975–0.988) | 0.967 (0.958–0.974) |
| Infection | 0.684 (0.607–0.753) | 0.933 (0.921–0.944) | 0.464 (0.400–0.530) | 0.972 (0.963–0.979) | 0.914 (0.901–0.926) |

Both classes are infrequent (blood presence 132/1,956, 6.7%; infection 152/1,956, 7.8%), so specificity and negative predictive value are correspondingly high. These are image-level classification results within the model-development corpus and are distinct from the clinician-adjudicated clinical outcomes of the study cohort. The model (Google Gemini 2.5 Flash) was fine-tuned and served on a managed cloud platform (Vertex AI); results are therefore reported from the platform’s held-out evaluation (Table A.2.9).
